# Efficient synthesis of oligofluoranthene nanorods with tunable functionalities†
†Electronic supplementary information (ESI) available: SEM images of OFA nanorods. See DOI: 10.1039/c5sc03041b


**DOI:** 10.1039/c5sc03041b

**Published:** 2015-09-17

**Authors:** Xin-Gui Li, Yaozu Liao, Mei-Rong Huang, Richard B. Kaner

**Affiliations:** a State Key Laboratory of Pollution Control and Resource Reuse , College of Environmental Science and Engineering , Tongji University , Shanghai 200092 , China . Email: adamxgli@yahoo.com ; Email: huangmeirong@tongji.edu.cn ; Fax: +86-21-65983869 ; Tel: +86-21-65983869; b Department of Chemistry & Biochemistry , California NanoSystems Institute , University of California , Los Angeles , California 90095 , USA . Email: kaner@chem.ucla.edu ; Fax: +1 310 206 4038 ; Tel: +1 310 825 5346; c State Key Laboratory for Modification of Chemical Fibers & Polymer Materials , College of Materials Science and Engineering , Donghua University , Shanghai 201620 , China; d Department of Materials Science & Engineering , University of California , Los Angeles , California 90095 , USA

## Abstract

Strongly fluorescence-emitting oligofluoranthene (OFA) nanorods are readily synthesized by a direct template-free chemical oxidative oligomerization of fluoranthene in nitromethane containing ferric chloride as an oxidant. The OFAs likely consist of five fluoranthene units containing cyclic pentamers with crystalline order and tunable electrical conductivity across 12 orders of magnitude. The OFA nanorods are heat-resistant materials and efficient precursors for macroporous carbon materials with high carbon yield in argon at 1100 °C. In particular, the optimal ring-like pentamer shows 12.2 times stronger cyan fluorescence-emission than recognized highly fluorescent fluoranthene under the same conditions, which makes the OFAs into ideal strong fluorescent emitters, tunable conductors, and high carbon-yield precursors for the preparation of sensors and carbon materials. These findings demonstrate an advance in the direct synthesis of oligomers from fused-ring aromatic hydrocarbons and provide a potential direction to optimize the synthesis and functionalities of wholly aromatic nanomaterials.

## Abstract 2

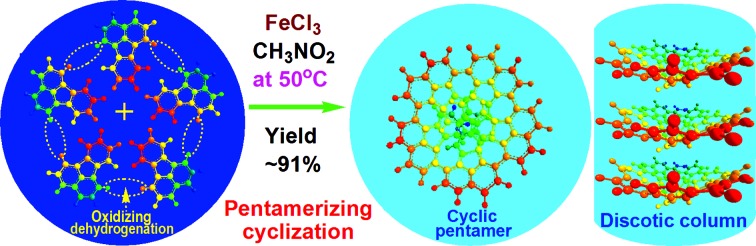
Strongly fluorescence-emitting oligofluoranthene nanorods efficiently synthesized by template-free oligomerization of fluoranthene in nitromethane demonstrate tunable conductivity and porous carbon formability.

## Introduction

Since conjugated polymers demonstrate versatile functionalities including unique energy migration across metal ions as a result of variations in absorption, emission, and redox properties, they have great potential for applications in highly sensitive sensors^1^,^2^ based on extraordinarily efficient energy transfer quenching and in polymer light emitting diodes. In comparison to conventional small molecule-based sensors, conjugated polymer chemosensors exhibit some important advantages due to signal amplification when subjected to external stimuli.^3^,^4^ As one of the important polycyclic aromatic hydrocarbons, fluoranthene (FA) has been used as a dopant for molecular crystals used in laser spectroscopy because of its strong fluorescent emission.^5^ Fluoranthene-based oligomers represent a new type of conjugated polymer that exhibits a low band gap, and novel electronic, optical and photovoltaic properties attributed to its unique ladder-type topology and highly delocalized π-conjugation.^6^ It is believed that oligofluoranthene (OFA) possesses better electronic and optoelectronic properties than that of the fluoranthene monomer which by itself is suitable for chemosensors.^7^ Recently we reported that OFA can emit strong fluorescence that gives rise to a unique superamplified quenching effect for the fabrication of advanced chemosensors for Fe(iii) and explosives such as picric acid.^8^ However, a systematic investigation on the scalable synthesis of functional OFA has been yet been carried out. To the best of our knowledge, only two papers are concerned with making OFA films and these were carried out by electropolymerization.^9^,^10^ Unfortunately, the OFA films prepared electrochemically have low electrical conductivity, limited synthetic yield, and poor thermal stability because of their irregular structures and low degree of conjugation.

On the other hand, porous carbon materials have attracted more attention because they hold great potential for important applications in fuel cells,^11^ lithium ion batteries,^12^,^13^ electronic capacitors,^14^,^15^ and adsorbents for environmental protection^16^,^17^ due to their low density, high thermal and electrical conductivity, high chemical and mechanical stability, and large specific surface area. A variety of methods such as template-synthesis,^18^ ultrasonic spray pyrolysis,^19^ solvent-assisting synthesis,^20^ and flash heating^21^ have been applied to prepare porous carbon materials. OFA as a highly aromatic oligomer has a theoretical carbon content as high as 96%. The thermal stability of OFA is high enough to prepare porous carbon because of its highly aromatic rings without any thermally unstable structures. Therefore, a new synthetic method for producing high-quality OFA nanomaterials for developing their applications as porous carbon precursors also holds great interest.

In this work, we explore a simple scalable method to synthesize OFA nanorods by using a direct chemical oxidative oligomerization of fluoranthene without any templates. The effect of oxidant species, oxidant/monomer ratio, polymerization temperature, and reaction time on the oligomerization yield, structure, morphology, properties, and functionalities of the OFA is explored. The as-synthesized OFA nanorods as precursors for macroporous carbon materials having widely tunable conductivities, ranging from 10^–9^ S cm^–1^ for virgin OFA to 10^–4^ S cm^–1^ upon iodine-doping and up to 100 S cm^–1^ upon carbonization, is systematically investigated. The fluorescence emission of the OFAs is optimized and controlled by adjusting the oxidant/monomer ratio and the OFA solution concentration. Chemical oxidative synthesis of the OFA nanorods directly from FA monomer is considered as a simple and effective route to make highly fluorescent organic substances and high carbon-yield precursors to porous carbon materials.

## Experimental section

### Synthesis of oligofluoranthene (OFA) nanorods

As a typical synthetic procedure for the OFA nanorods, 1 mmol (0.21 g) of FA monomer and 9 mmol of anhydrous FeCl_3_ oxidant were dissolved in 5 mL of CH_3_NO_2_, respectively. The two solutions were kept at 50 °C in a water bath for 30 min and then the FeCl_3_ solution was slowly added into the FA solution during magnetic stirring. The mixture was further magnetically stirred for 15 h at 50 °C to complete the oligomerization. Note that upon adding the first drop of the oxidant solution into the monomer solution, the light-yellow monomer solution quickly turned orange. The color then gradually became darker as more oxidant solution was added and turned completely black after finishing the addition of the FeCl_3_ solution. Virgin nanorods were obtained after purification with ethanol and DI water by centrifugation. Dedoped nanorods were prepared by further washing successively with 1.0 M HCl, 1.0 M NaOH and DI water several times at 50 °C in order to remove the residual monomer, oxidant and their HCl/NaOH aqueous soluble byproducts. Finally, the pure dedoped OFA particles were obtained after drying at 80 °C for 48 h. Dedoped OFA particles were doped by iodine vapor as follows: OFA/iodine (3/97) particles were kept in a sealed vessel at 80 °C under atmospheric pressure for two days, during which the OFA and iodine particles in the vessel were not allowed to contact each other.

### Measurements

UV-vis spectra of dedoped OFA particles in *N*-methylpyrrolidone (NMP) and concentrated H_2_SO_4_ were recorded on a Perkin-Elmer Lambda 25 UV-vis spectrophotometer. ATR-FTIR spectra were obtained with a Nicolet Magna-IRTM 550. Raman spectra of the solid powder of the OFA and PFA were taken using a British Renishaw inVia Raman Microscope (Bert) with a 785 nm red laser pumped with a solid state diode. The molecular weight of the soluble part of the OFA nanorods was obtained on a matrix-assisted laser desorption time-of-flight mass spectrometer (MALDI-TOF-MS) spectrum using a Voyager DE STR MALDI-TOF mass spectrometer with 2,5-dihydroxybenzoic acid as the matrix. ^1^H-NMR spectra were taken on a Bruker ARX-500 and 400 spectrometer using dimethyl sulfoxide (DMSO)-D_6_ and CDCl_3_ as solvents. X-ray diffraction (XRD) patterns were scanned on a Philips X'pert Pro powder diffractometer using copper-monochromatized CuKα radiation (*λ* = 0.154178 nm). Morphologies of OFA and carbon-based materials were observed on a JEOL JSM-6700 field emission scanning electron microscopy (SEM), whose samples were prepared by dropping the ethanol dispersion of the particles onto silicon wafers and then gold sputtering. Fluorescence spectra of FA and OFA solutions in NMP were acquired on a QM-6SE PTI fluorescence spectrometer. Thermogravimetric (TG) analysis was carried out on a Perkin Elmer TGA Pyris 1 in argon at a heating rate of 15 °C min^–1^ from room temperature to 1100 °C. The carbon materials made from TG measurements were imaged by a JEOL JSM-6700 SEM without gold sputtering. The bulk electrical conductivity of an approximately 0.5 mm-thick pressed disk of OFA particles was measured using a two-probe method with an area of 0.785 cm^2^ at room temperature.

## Results and discussion

### Synthesis of OFA particles

#### Optimization of the oxidants

Three representative oxidants with different standard reduction potentials (RP), such as concentrated H_2_SO_4_ (RP = 0.17 V *vs.* SCE), FeCl_3_ (0.771 V *vs.* SCE), and ammonium persulfate (2.01 V *vs.* SCE) were chosen to oxidatively oligomerize the FA monomer.

**Fig. 1:**
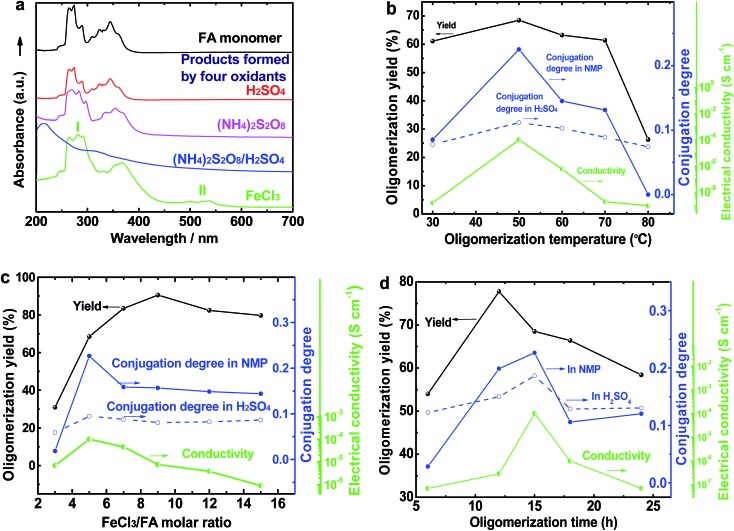
(a) UV-vis spectra of FA and OFAs synthesized with different oxidants. The effects of (b) oligomerization temperature, (c) FeCl_3_/FA ratio, and (d) oligomerization time on the oligomerization yield, conjugation degree, and I_2_-doping conductivity of the OFAs.

The UV-vis spectrum of an FA solution in NMP does not display any significant absorption at wavelengths above 400 nm (Fig. 1a). The reaction products obtained using only H_2_SO_4_ or ammonium persulfate present very similar UV-vis absorptions to that of the monomer, indicating that these two oxidants do not lead to a successful synthesis of OFA because of their too low or too high standard RPs, respectively. A combination of H_2_SO_4_ and ammonium persulfate results in some kind of oxidative oligomerization of FA monomer because the product exhibits a completely different UV-vis spectrum with a weak absorbance tailing at long wavelengths up to 600 nm. It is interesting to note that when the solution FeCl_3_/CH_3_NO_2_ was applied as an oxidant, the reaction instantly turned black, finally leading to solid dark particles. By continually adding FeCl_3_ solution, the reaction color became darker with black flocculates deposited on the bottom of the reaction vessel. Brownish red precipitates were obtained after washing with alcohol and water. The product prepared by FeCl_3_ shows new UV-vis absorptions at 499 and 537 nm associated with large π-conjugation among different FA units, signifying the occurrence of the oxidative oligomerization of FA.^8^,^22^ Furthermore, the conjugation degree of the OFA can be quantitatively evaluated by defining the relative UV-vis absorbance ratio of Band II and Band I, as shown in Fig. 1b–d. Clearly, FeCl_3_ is the best oxidant because OFA synthesized with FeCl_3_ possesses the highest degree of conjugation. In fact, FeCl_3_ is not only an oxidant, but also a Lewis acid. This chemical has been successfully used as an oxidant for C–C coupling reactions with other conjugated molecules including benzene, pyrene, pyrrole, and triphenylene.^7^,^23^,^24^ Therefore, FeCl_3_ has been chosen as an optimal oxidant for the following investigation.

#### Optimization of the oligomerization temperature

**Fig. 2:**
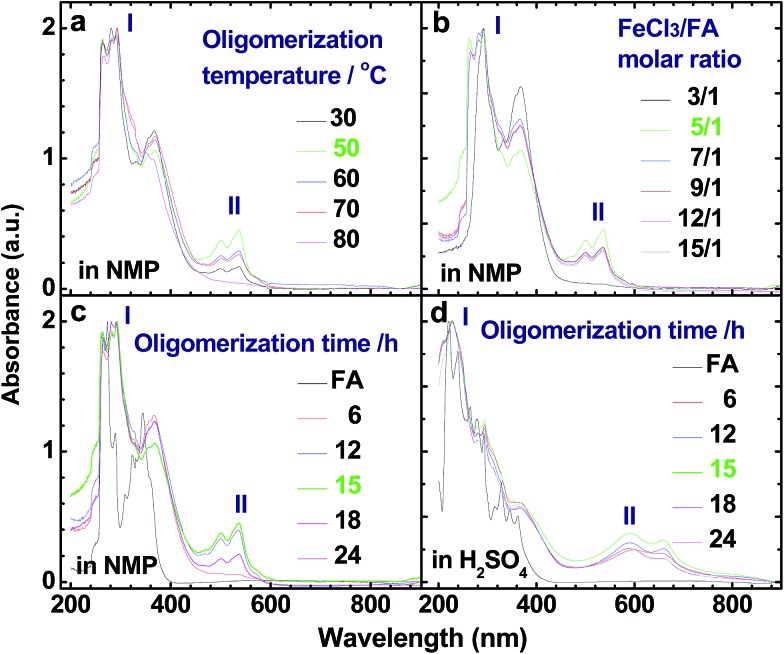
UV-vis spectra of OFAs synthesized under different conditions: (a) oligomerization temperature, (b) FeCl_3_/FA molar ratios, and (c and d) oligomerization times using (c) NMP and (d) concentrated H_2_SO_4_ as test solvents, respectively.

Fig. 1b and 2a illustrate the influence of oligomerization temperature on the synthetic yield and conjugation degree of OFA at a fixed FeCl_3_/FA ratio of 5/1 and oligomerization time of 15 h. By elevating the temperature from 30 to 80 °C, both the oligomerization yield and π-conjugation degree increase first and then decrease, exhibiting maxima of 68.5% and 0.23 at 50 °C, respectively. At 80 °C, however, the UV-vis spectrum no longer shows any significant π-conjugation absorption. Meanwhile, the oligomerization yield is the lowest dropping down to 26.3%. This signifies that 50 °C is the optimal temperature to synthesize large π-conjugated OFA directly from FA. A possible reason is that too low a temperature does not supply enough reaction activation energy for the oligomerization, while too high a temperature leads to too fast an oligomerization rate, thus degrading the regularity of the OFA macromolecular structure. This also explains why the oligomerization temperature has a great impact on the conductivity of the OFA, which will be discussed later.

#### Optimization of the oxidant/monomer ratio

The influence of FeCl_3_/FA ratio on the oligomerization yield and π-conjugation degree at a fixed temperature/time of 50 °C/15 h is presented in Fig. 1c and 2b. When the FeCl_3_/FA molar ratio was 3/1, the as-synthesized OFA does not seem to show any remarkable large π-conjugated characteristics because of the absence of UV-vis absorptions at wavelengths at or above 400 nm. When the FeCl_3_/FA molar ratio was increased to 5/1, the OFA obtained exhibits two strong π-conjugation absorptions at 501 and 537 nm and thus, the highest conjugation degrees of 0.23 in NMP and 0.09 in concentrated H_2_SO_4_. However, the maximum oligomerization yield (90.5%) occurred at an oxidant/monomer molar ratio to 9/1. That is to say, the OFA has the most conjugated structure and the highest oligomerization yield at FeCl_3_/FA molar ratios of 5/1 and 9/1, respectively. This implies that too little oxidant could not produce sufficient reactive sites for oligomerization, but too much oxidant weakens the conjugation degree due to over-oxidation. Undoubtedly, the FeCl_3_/FA molar ratio of 5/1 is the best for the synthesis of the OFA with the optimal π-conjugated structure. The FeCl_3_ dosage is much higher than that for oxidative dehydrogenation oligomerization of benzene^23^ by CuCl_2_/AlCl_3_ (ref. 25) since there are more dehydrogenation sites in FA than benzene.

#### Optimization of the oligomerization time

The influence of the oligomerization time on the synthetic yield and OFA conjugation degree at a fixed temperature of 50 °C and FeCl_3_/FA ratio of 5/1 is depicted in Fig. 1d and 2c and d. With increasing oligomerization time from 6 to 24 h, the maximum yield of 77.8% and highest conjugation degree of 0.23 in NMP and 0.19 in concentrated H_2_SO_4_ appear at 12 h and 15 h, respectively. Generally, the oxidative polymerization of aromatic hydrocarbons has three typical reaction stages. In case of FA oligomerization in the presence of H^+^[FeCl_3_(CH_2_NO_2_)]^–^, the FA monomers would be quickly turned into highly reactive cations. With increasing oligomerization time, the cations would couple to produce dimeric intermediates. With continuously lengthening time, the oxidative dehydrogenation, oligomerization, and cyclization among the monomer/dimer/tetramer and their cations finally results in the formation of OFA oligomers, as discussed below. It should be noted that too long a reaction time would inevitably cause over-oxidation of the oligomers formed, decreasing both yield and π-conjugation to some extent. Regardless of the similar trend of conjugation degree in NMP and H_2_SO_4_ with time, the OFAs synthesized under different oligomerization times exhibit longer wavelength UV-vis absorptions at 590 and 660 nm in H_2_SO_4_ compared to 501 and 537 nm in NMP, respectively. This may be attributed to the formation of polarons in OFA due to its large π-conjugation structure originating from the doping effects of H_2_SO_4_.^26^,^27^ Clearly, the optimal synthetic conditions of OFA in CH_3_NO_2_ are as follows, oligomerization temperature: 50 °C, time: 15 h, and FeCl_3_/FA molar ratio: 5/1.

### Structure of the oligofluoranthenes

#### UV-vis spectra

Fig. 2c and d show UV-vis spectra for FA (black line) and the soluble part of the OFA nanorods synthesized under the optimal conditions (green line). The OFA demonstrates (1) two strong bands at 265–300 nm associated with the π–π* transition of the FA units that are different from those of the FA monomers,^28^ (2) two additional bands at 501/537 nm in NMP and 590/660 nm in H_2_SO_4_ attributed to the n–π* transition of the quinoid structure of the FA units, and (3) an absorbance tail extending to 600 nm in NMP and 800 nm in H_2_SO_4_. Obviously, the absorbance at 501 nm and longer wavelengths signifies a large π-conjugated structure along the molecular rings of the OFAs. In particular, the OFAs obtained here achieve much stronger absorptions in a much longer wavelength range than the OFA synthesized by electropolymerization,^10^ indicating that the chemical oxidative oligomerization is more effective at synthesizing OFA with a large π-conjugated structure.

#### IR spectra

**Fig. 3:**
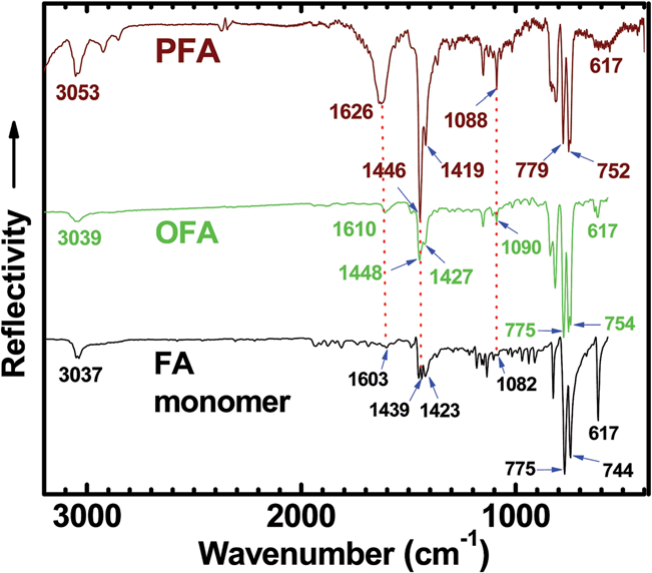
FTIR spectra of FA monomer, OFA synthesized under optimal conditions, and polyfluoranthene (PFA) particles synthesized in *n*-hexane/nitromethane volume ratio of 3/2 with FA/FeCl_3_ molar ratio of 7. The absorption intensity is renormalized to the 775–752 cm^–1^ band intensity.

An IR spectrum of FA monomer powder shows strong absorptions at 775 and 744 cm^–1^ due to FA out-of-plane bending modes (*γ*_Ar–H_),^8^,^29^,^30^ as shown in Fig. 3. The IR spectrum of OFA nanorods (with moderate molecular weight) synthesized under optimal conditions shows slightly different strong absorptions at 775 and 754 cm^–1^, while the IR spectrum of PFA particles with much higher molecular weight shows strong absorptions at 779 and 752 cm^–1^.^31^ If using the FA out-of-plane bending modes around 775–754 cm^–1^ as an internal standard, the three absorption bands at *ca.* 1090, 1448 and 1610 cm^–1^ due to framework vibrations of FA units become stronger and broader when changing samples from FA monomer to OFA to PFA, *i.e.*, increasing their molecular weight. This can be attributed to the diversification of aromatic structures because of the C–C coupling caused by dehydrogenation during the oxidative oligomerization and/or polymerization. It should be noted that the weak absorbance of FA at *ca.* 3037 cm^–1^, corresponding to stretching vibrations of aromatic hydrogen (Ar–H),^8^,^32^ shifts to a higher wavenumber (3039 cm^–1^) for OFA and to a much higher wavenumber (3053 cm^–1^) for PFA, again owing to a steadily enhanced molecular weight from FA to OFA to PFA. In contrast, the sharp and strong absorbance at 617 cm^–1^ is a characteristic band of FA monomer because this band becomes much weaker for OFA and PFA with much higher molecular weights. It appears that the intensity of absorption bands at *ca.* 1090, 1448 and 1610 cm^–1^ could be used to semi-quantitatively evaluate the molecular weight when the absorption intensity is renormalized to the 775–752 cm^–1^ band intensity because the absorption intensity at 775–752 cm^–1^ hardly changes with going from FA to OFA to PFA. In other words, the stronger absorption bands at *ca.* 1090, 1448 and 1610 cm^–1^ imply higher molecular weight for the FA oligomers and/or polymers.

#### Raman spectra

**Fig. 4:**
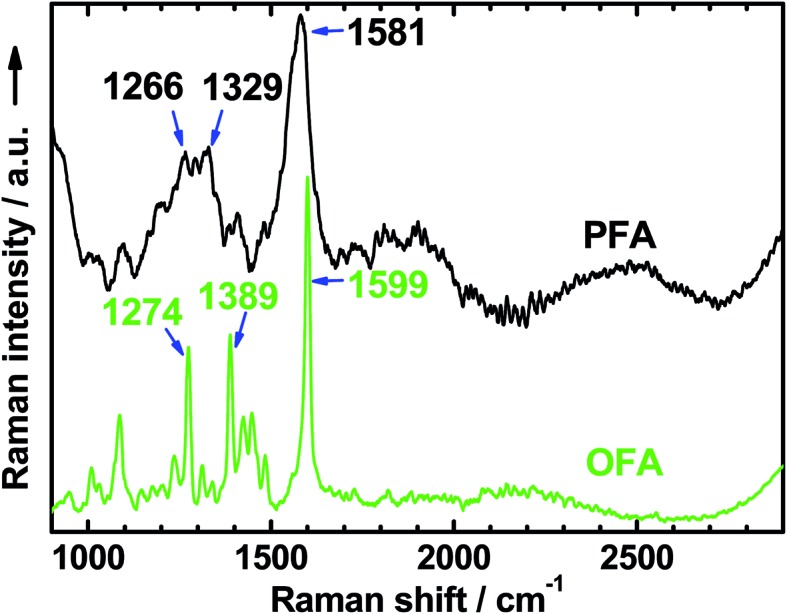
Raman spectra of OFA and PFA synthesized with the same FeCl_3_ oxidant in CH_3_NO_2_ and CH_3_NO_2_/hexane as the reaction media, respectively.

The Raman spectrum of the solid nanorods of OFA in Fig. 4 shows several characteristic Raman bands centered at 1599, 1389, 1274, 1448, 1087, and 1425 cm^–1^ (from stronger to weaker), corresponding to the Raman vibrations of the FA units. These Raman bands are much sharper than those of polyfluoranthene (PFA),^31^ but broader than those of the FA monomer.^33^ Moreover, these Raman bands generally shift to higher wavenumbers compared to polyfluoranthene (PFA), but lower wavenumbers when compared to the FA monomer. All of these Raman spectral variations with PFA, OFA, and FA monomer contribute to the significant variation of their molecular weight from higher to lower. That is to say, the OFA has much lower molecular weight than PFA, but higher molecular weight than the FA monomer.

#### Elemental analysis and MALDI/TOF MS and NMR spectra

Elemental analysis results of three typical OFAs synthesized with three FeCl_3_/FA molar ratios are summarized in Table 1.^8^ It can be seen that the total C/H content depends on the FeCl_3_/FA ratio, *i.e.*, the OFAs formed at the FeCl_3_/FA molar ratios of 3/1, 5/1, and 7/1 have, respectively, total C/H contents of 90.92, 98.08, and 93.38 wt%. That is to say, the OFA formed at the FeCl_3_/FA molar ratio of 5/1 is the purest, which is likely one of the main reasons why this specific OFA emits the strongest fluorescence as discussed below. The possible molecular structure inferred based on the C/H ratio is also provided in Table 1. It appears that the oligomers form in a ring pentamer containing five FA units. The chemical bonds between any two neighboring FA units are sometimes greater than one at the FeCl_3_/FA molar ratios of 3/1 and 7/1. The OFA formed at the FeCl_3_/FA molar ratio of 5/1 has the most regular 3D ring configuration that could result in the largest degree of π-conjugation as shown in Fig. 1c and 2b. Two types of OFAs obtained at FeCl_3_/FA molar ratios of 3/1 and 5/1 exhibit similar 400 MHz ^1^H-NMR spectra in CDCl_3_. The OFA obtained at the FeCl_3_/FA molar ratio 5/1 demonstrates higher resolution possibly due to its better structural regularity and lower aromaticity revealed in Table 1 than that at the FeCl_3_/FA molar ratio of 3/1. A similar higher resolution spectrum has also been achieved for the OFA obtained at the FeCl_3_/FA molar ratio of 3/1 by using DMSO-d_6_ as the solvent at 500 MHz rather than CDCl_3_ at 400 MHz. Note that no high quality NMR spectra could be obtained for the OFAs synthesized at FeCl_3_/FA molar ratios of 7/1 and 9/1 because of their relatively low solubilities in CDCl_3_.

**Table 1:** Elemental analysis of the OFA nanorods synthesized with three FeCl_3_/FA molar ratios

FeCl_3_/FA molar ratio	Experimental C/H/N (wt%)	Element formula	Oligomerization reaction and synthesized OFA structure	3D structure	
				Top view of cylindrical bonds	Side view of cylindrical bonds
					Side view of sticks
3/1	87.70/3.22/0.15	C_16_H_6.99_	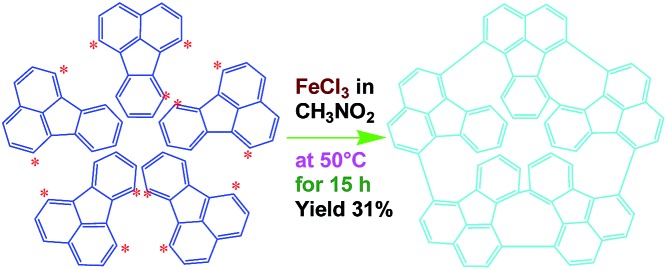	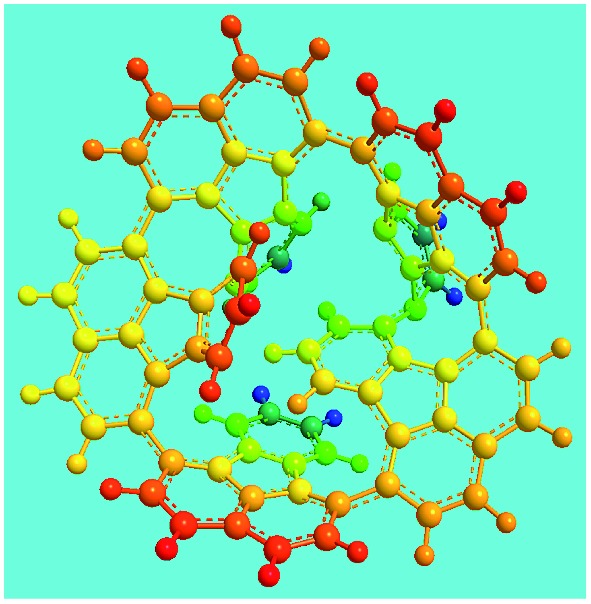	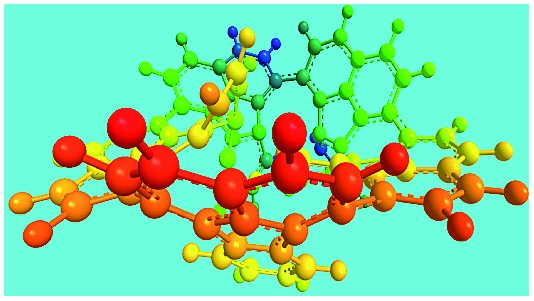
		(CH_3_NO_2_)_0.02_			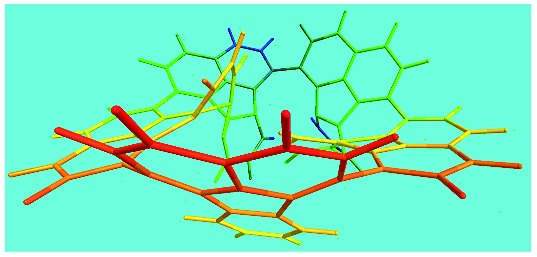
5/1	94.17/3.91/0.06	C_16_H_7.95_	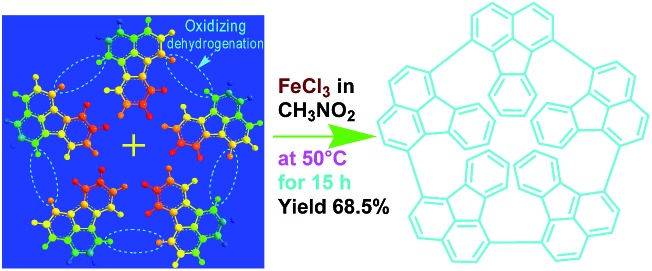	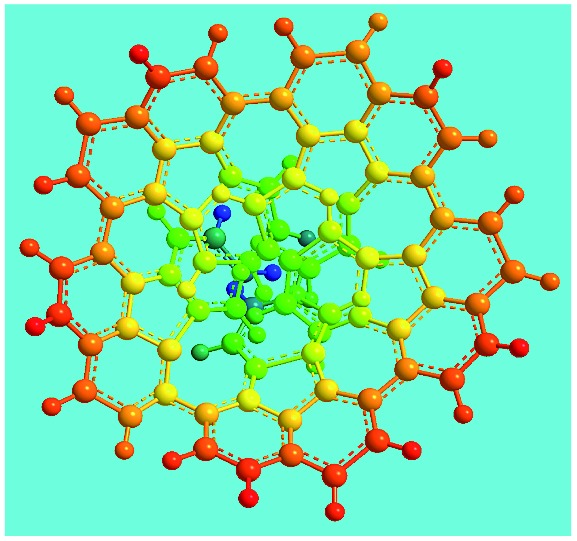	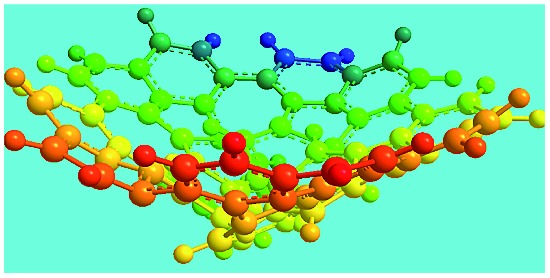
		(CH_3_NO_2_)_0.01_			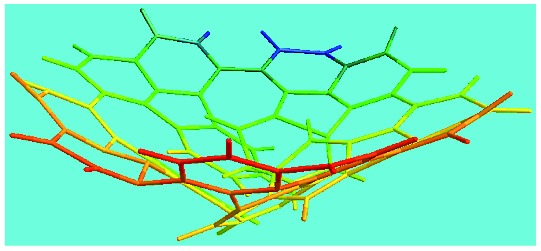
7/1	90.07/3.31/0.12	C_16_H_7.01_	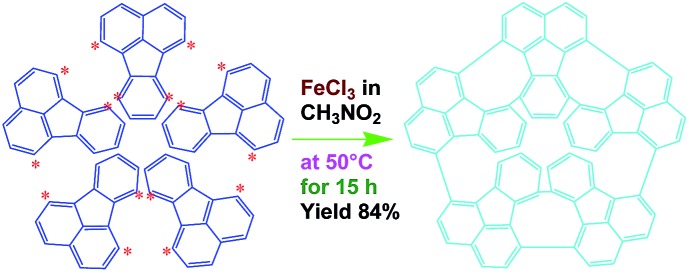	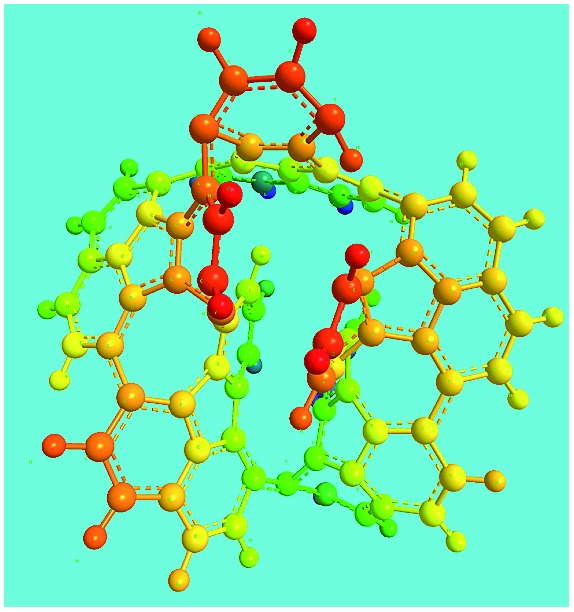	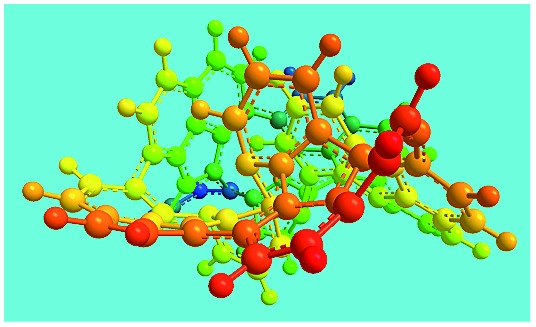
		(CH_3_NO_2_)_0.02_			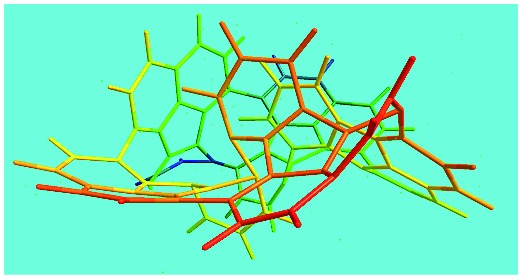

**Fig. 5:**
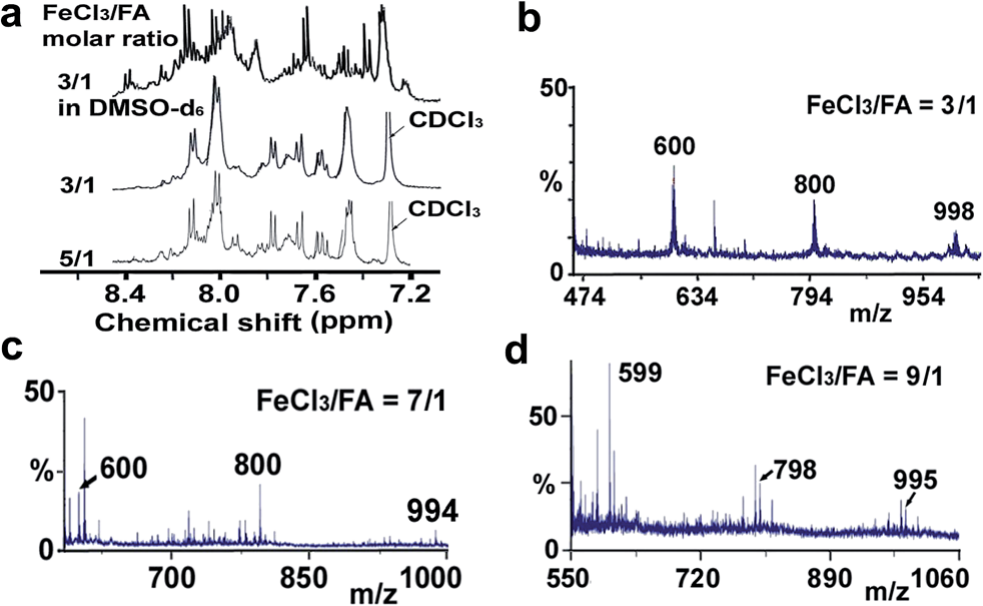
(a) ^1^H-NMR spectra in DMSO-d_6_ at 500 MHz and CDCl_3_ at 400 MHz and (b–d) MALDI/TOF MS spectra of the OFAs synthesized with three representative FeCl_3_/FA molar ratios.

Moreover, these molecular structures have been substantially confirmed by their MALDI/TOF MASS spectra shown in Fig. 5. The OFA obtained at the FeCl_3_/FA molar ratio of 3/1 in Fig. 5a probably consists of a mixture of two isomers as given in Table 1 since each of its characteristic peaks is composed of a few substances with a very close mass, while the OFA obtained at the FeCl_3_/FA molar ratios of 7/1 and 9/1 in Fig. 5b and c may contain some minor impurities besides the major 5-FA unit-containing oligomers likely because more oxidants can lead to over-oxidation of the OFA as discussed earlier. These imply that the molecular structure of the OFAs produced depends on the synthetic conditions.

##### Oxidative pentamerization mechanism of FA

**Scheme 1:**
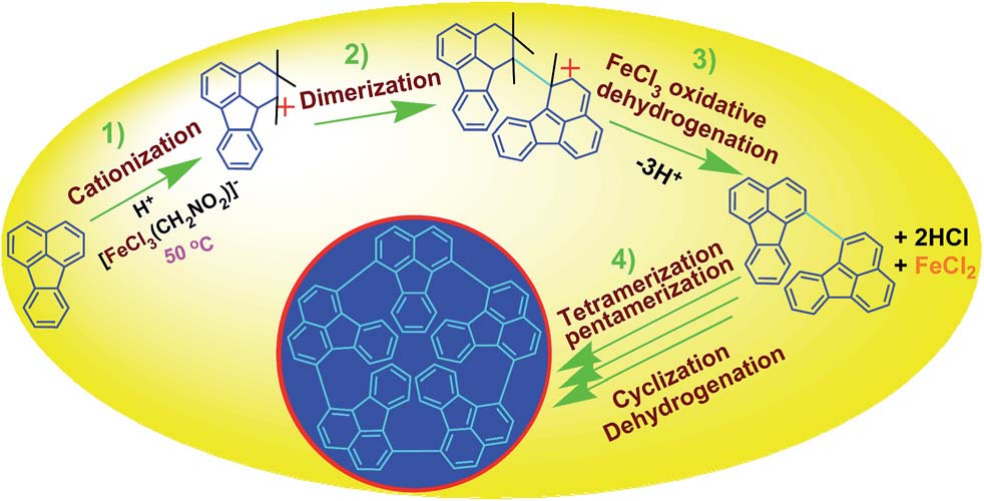
Proposed mechanism of direct cationic oxidative pentamerization of FA in CH_3_NO_2_.

The possible mechanism for the direct cationic chemical oxidative pentamerization of FA in CH_3_NO_2_ was proposed in Scheme 1.

FeCl_3_ can react with CH_3_NO_2_ to generate *in situ* the H^+^[FeCl_3_(CH_2_NO_2_)]^–^ complex, which initiates the cationic oxidative pentamerization of FA just like the polymerization of benzene and pyrene by FeCl_3_.^7^,^23^ The FA pentamerization likely consists of the following steps: (1) the cationization of FA monomers and the formation of FA carbonium ions by H^+^, (2) the dimerization of FA carbonium ions with concomitant formation of a dimeric cation, (3) the oxidative dehydrogenation of FA dimeric carbonium ions by FeCl_3_, (4) the tetramerization/pentamerization, cyclization, and dehydrogenation among active FA dimer, trimer, and pentamer carbonium ions. The constant decline of both the open-circuit potential and the pH of the FA oligomerization solution containing FeCl_3_ oxidant with reaction time supports this pentamerization mechanism because of a fast consumption of the oxidant to produce cation oligomerization and also HCl as a byproduct. In short, FA monomers likely undergo cationization, coupling, dehydrogenation, and cyclizing pentamerization to give the resulting OFAs.

#### XRD diffractograms

**Fig. 6:**
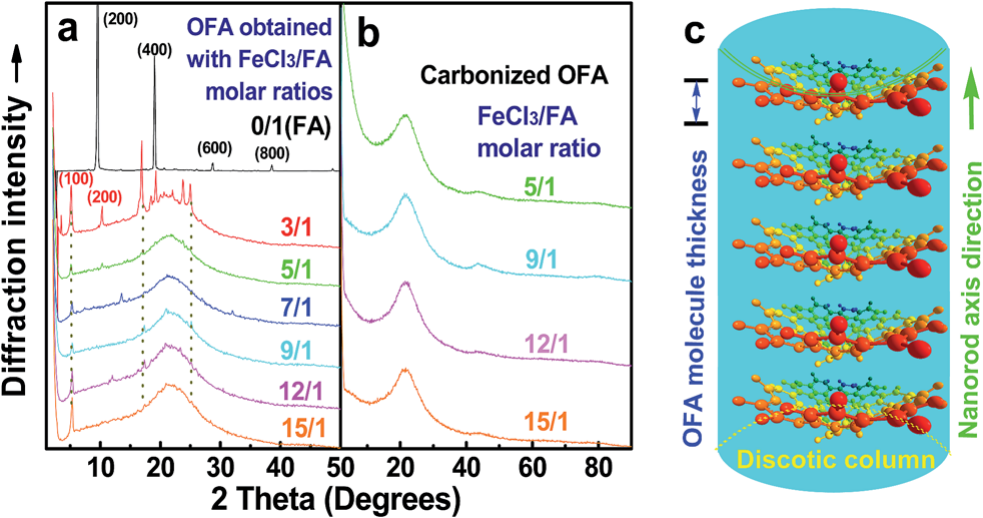
XRD patterns of (a) pure FA monomer and OFAs synthesized with different FeCl_3_/FA ratios; (b) OFA-based carbon materials; and (c) discotic column consisting of the conical OFA molecules.

Fig. 6a shows wide-angle X-ray diffractograms of FA and OFA nanorods synthesized with various oxidant/monomer ratios. Compared with the highly crystalline suprastructure of FA demonstrating an extremely strong diffraction peak at 2*θ* 9.51° (*d*_200_ 0.929 nm),^8^ a strong peak at 2*θ* 19.03° (*d*_400_ 0.466 nm), and two weak peaks at 2*θ* 28.76° (*d*_600_ 0.310 nm) and 38.59° (*d*_800_ 0.233 nm), the OFA nanorods exhibit a characteristic broad diffraction peak centered at 21.5° (d 0.413 nm) assigned to an amorphous suprastructure of the OFA nanorods regardless of the FeCl_3_/FA ratio used for their synthesis. Obviously, the chemical oxidative polymerization makes crystalline FA monomer become non-crystalline OFA with much higher molecular weight, but much lower supramolecular order of the FA units. Note that all OFAs exhibit a new sharp diffraction peak at 2*θ* 5.16° (*d*_100_ 1.711 nm), a moderate peak at 10.31° (*d*_200_ 0.857 nm), and the strongest peak at 16.60° (*d* 0.534 nm). The *d*_100_ diffraction intensity decreases first and then increases with increasing FeCl_3_/FA molar ratio from 3/1 to 15/1, demonstrating the minimal intensity at an FeCl_3_/FA molar ratio of 9/1. That is to say, the OFA at the FeCl_3_/FA ratio of 9/1 has the least crystalline order. These diffraction peaks should arise from crystalline ordered structures in OFA nanorods. One more moderate peak at 24.95° (*d* 0.356 nm) could be related to the intracolumnar order. It appears that these diffraction peaks reveal a molecular crystalline arrangement in which the OFA rings are nearly perpendicular to the OFA nanorod axis as shown in Fig. 6c. The normal line direction of the *d*_100_ and *d*_200_ crystal facets is likely perpendicular to the nanorod axis direction. The fact that the OFAs have smaller *d*_200_-spacing than FA provides evidence that the OFA molecules exhibit 3D conical configurations with smaller thicknesses. There are no diffraction patterns of FeCl_3_/FeCl_2_ found in the OFAs, which implies that the OFA nanorods have high purity; this is very important to their potential applications as both fluorescent emitters and chemosensors.

### Morphology

**Fig. 7:**
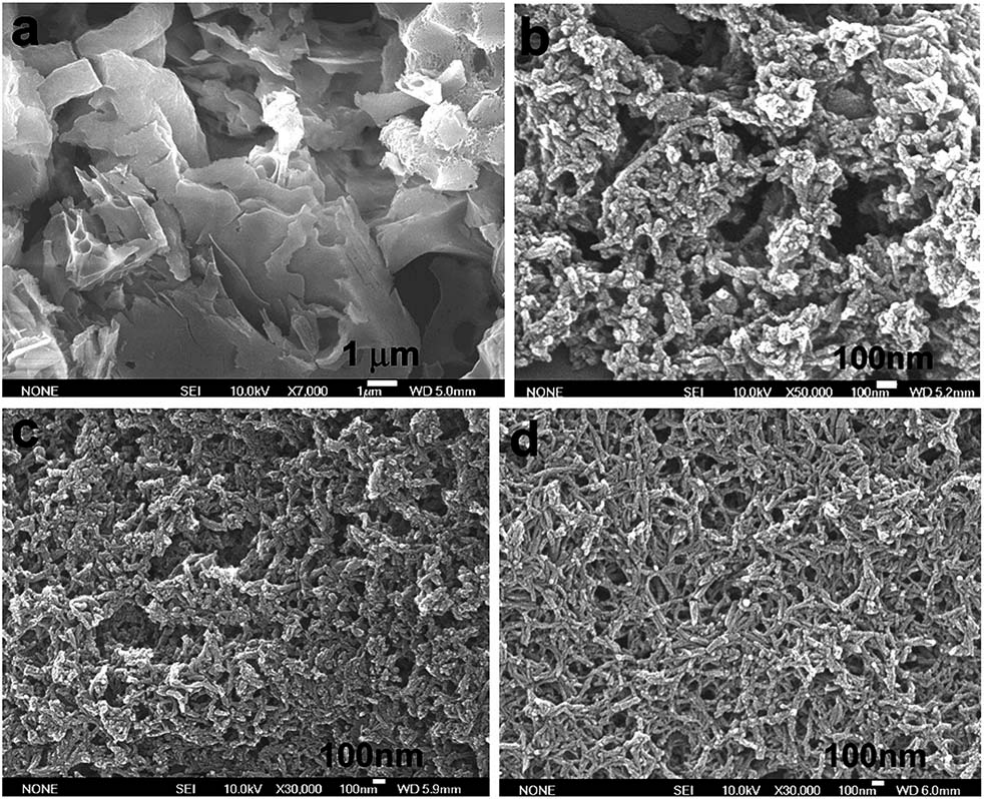
SEM images of OFA particles and nanorods synthesized with FeCl_3_/FA molar ratios: (a) 3/1, (b) 5/1, (c) 9/1, and (d) 12/1.

Fig. 7 shows SEM images of the OFA nanorods synthesized with four different oxidant/monomer ratios. When an FeCl_3_/FA molar ratio of 3/1 is used, the OFAs formed look like large flakes and needles with smooth surfaces. By increasing the oxidant/monomer molar ratio to 5/1, 9/1, and up to 12/1, the obtained OFAs mainly appear as rod-like nanostructures with diameters around 35, 38, and 40 nm, respectively, and lengths greater than 300 nm. It appears that the morphology of the OFAs significantly depends on the FeCl_3_/FA ratio, which can be confirmed by five more sharp diffraction peaks for the OFA formed at the FeCl_3_/FA molar ratio of 3/1 when compared to the other OFAs at FeCl_3_/FA molar ratios between 5/1 and 12/1. It has been reported that many electrically conducting polymers or oligomers such as poly(*o*-anisidine),^34^ poly(azomethine),^35^ poly(3-hexylthiophene),^36^ and thiophene/phenylene co-oligomer^37^ also self-assemble into one-dimensional nanostructures through strong π–π interactions between the packed molecules. Analogously, the π–π stacking in the OFAs with conical configurations could be one of the reasons for the formation of one-dimensional nanorods.^8^ A significant red-shift of 192 nm in the UV-vis and fluorescence spectra of the OFAs can be attributed to the strong π–π stacking.

### Solubility and solvatochromism

Table 2 shows the solubility and solvatochromism of FA and OFAs synthesized with various FeCl_3_/FA ratios. Although the FA is soluble in most organic solvents including HCOOH, NMP, DMSO, dimethyl formamide (DMF), CH_3_CN, tetrahydrofuran (THF), and *n*-hexane, most of the OFAs are soluble in highly polar NMP with high polarity index, but partly or slightly soluble in DMSO, DMF, H_2_SO_4_ and HCOOH, which is in accordance with other conducting polymers reported earlier.^38^–^41^ Meanwhile, the obtained OFAs are insoluble, but dispersible in CH_3_CN, THF, and *n*-hexane. This signifies that the OFAs have higher chemical resistance than FA likely due to their higher molecular weight and thus larger π-conjugated structures. The OFAs synthesized under optimal conditions show the lowest solubility and the darkest color in various solvents due to the highest degree of π-conjugation. Furthermore, the optimal OFA displays a unique solvatochromism including yellow, green, red, dark green, and gray in different solvents associated with the variation of the conformations of OFA molecules. The OFAs synthesized with different FeCl_3_/FA ratios all demonstrate longer wavelength UV-vis absorptions at 660–663 nm in H_2_SO_4_ when compared to 535–537 nm in NMP, which explains that the unique solvatochromism is due to their different absorptions.

**Table 2:** Solvatochromism and thermal degradation of FA and OFAs

FeCl_3_/FA molar ratio	Solubility^*a*^ (solution color^*b*^, UV-vis maximum absorption wavelength [nm])								Thermal degradation parameters			
	Hexane	THF	CH_3_CN	DMF	DMSO	NMP	HCOOH	H_2_SO_4_	*T*_d_ ^*c*^ (°C)	*T*_dm_ ^*d*^ (°C)	(d*α*/d*t*)_m_ (% min^–1^)	Carbon yield at 1100 °C (%)
0/1(FA)	S(y)	S(c)	S(y)	S(c)	S(c)	S(c, 345)	S(y)	PS(g, 362)	211	344	32.4	0
3/1	ID(g)	SS(y)	SS(br)	MS(y)	MS(r)	S(r, 535)	SS(gr)	PS(dg, 660)	NA	NA	NA	NA
5/1	ID(br)	ID(r)	ID(br)	PS(r)	PS(r)	S(r, 537)	SS(gr)	PS(dg, 663)	384	497	5.9	23.2
7/1	ID(br)	ID(r)	ID(br)	PS(r)	PS(p)	S(r, 536)	SS(gr)	PS(dg, 662)	419	528	1.6	66.8
9/1	ID(br)	ID(r)	ID(br)	PS(r)	PS(p)	S(r, 536)	SS(gr)	PS(dg, 662)	446	633	1.2	77.6
12/1	ID(br)	ID(r)	ID(br)	PS(r)	PS(p)	S(r, 536)	SS(gr)	PS(dg, 662)	445	628	2.9	28.7
15/1	ID(br)	ID(r)	ID(br)	PS(r)	PS(p)	S(r, 536)	SS(gr)	PS(dg, 662)	425	604	3.3	29.2

^*a*^ID = insoluble but dispersible; MS = mainly soluble; PS = partially soluble; S = soluble; SS = slightly soluble.

^*b*^br = brownish red; c = colorless; dg = dark green; g = green; gr = gray; p = pink; r = red; y = yellow.

^*c*^The temperature corresponding to 2% weight loss.

^*d*^The temperature corresponding to the maximal weight-loss rate.

### Electrical conductivity

As compared with electrically insulating FA, the virgin OFAs are powders having conductivities ranging from 10^–11^ and 10^–7^ S cm^–1^. The conductivity can be enhanced by 3 orders of magnitude (to 10^–4^ S cm^–1^) by iodine doping. The effect of oligomerization temperature, FeCl_3_/FA molar ratios, and oligomerization time on the conductivity of iodine-doping OFA is summarized in Fig. 1b–d. With increasing FeCl_3_/FA molar ratio from 3/1 to 15/1, the conductivity increases first and then declines, exhibiting a maximum of 1.0 × 10^–4^ S cm^–1^ at 5/1, likely because too high an FeCl_3_/FA ratio severely impairs the conductivity due to over-oxidation of the OFA. Meanwhile, it can be seen that the maximum conductivity occurs at the optimal oligomerization temperature and time of 50 °C and 15 h, respectively. This is due to the maximum doping levels and the maximum degree of π-conjugation that is confirmed by the UV-vis spectra shown in Fig. 2. Thus, it is concluded that iodine vapor doping is an effective method to significantly enhance the conductivity of the OFAs, which is associated with enhancement of the charge carrier concentration.^7^,^23^

### Thermal degradation of the OFAs for preparation of porous carbon

**Fig. 8:**
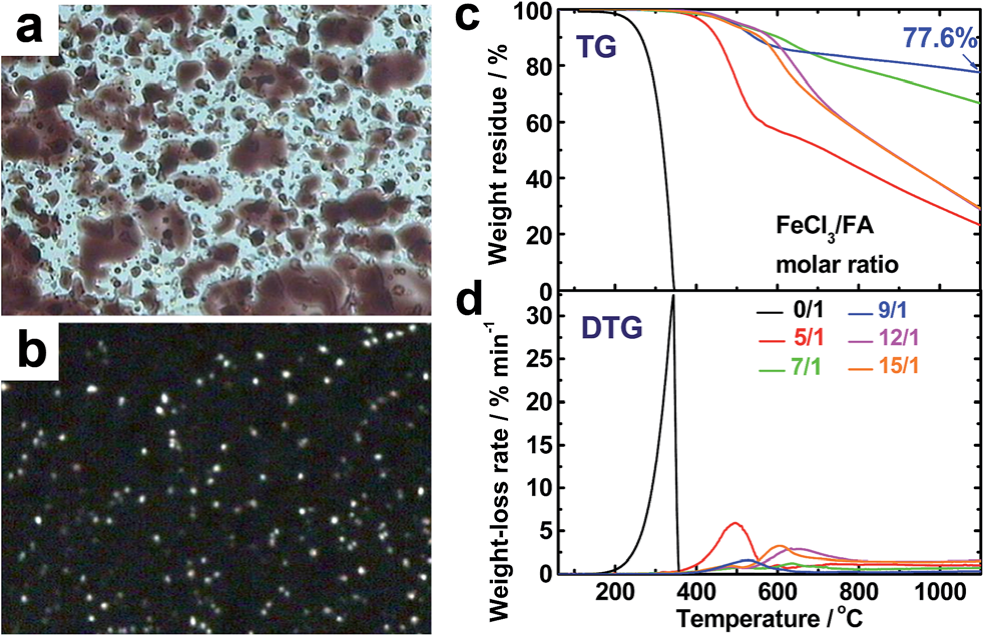
Optical microscope images of OFAs (after heating to 300 °C) under (a) sunlight and (b) polarized light. (c) TG and (d) DTG curves of FA and the OFAs synthesized with different FeCl_3_/FA ratios.

The virgin OFA powders do not exhibit optical birefringence at room temperature under a polarized light microscope, implying that they are optically isotropic. The OFA powders melt at 300 °C and the melt exhibits optically anisotropic order because optical birefringence appears, as shown in Fig. 8a and b. This optical birefringence disappears after the OFA sample thoroughly melts or decomposes at higher temperatures. It appears that the OFAs are thermotropic liquid crystals because they can be considered as wholly aromatic discotic molecules as schematized in Table 1 and Fig. 6c. The thermal degradation of the OFAs synthesized with different oxidant/monomer ratios was quantitatively studied by TG as presented in Fig. 8c and d and Table 2. The FA monomer loses its entire weight over a temperature range from 200 to 346 °C, but the virgin OFAs in argon do not demonstrate any significant weight loss below 355 °C, further confirming that the synthesized OFAs have much higher molecular weights than the FA monomer. Moreover, by increasing the FeCl_3_/FA molar ratio from 5/1 to 15/1, the thermal degradation temperatures (*T*_d_ and *T*_dm_) and char yield at 1100 °C in argon first increase and then decrease, demonstrating maximum degradation temperatures and char yield at the FeCl_3_/FA ratio of 9/1. In contrast, the maximum weight-loss rate, (d*α*/d*t*)_m_, first decreases and then increases with increasing FeCl_3_/FA ratio from 5/1 to 15/1, demonstrating a minimum (d*α*/d*t*)_m_ at the FeCl_3_/FA molar ratio of 9/1. These results signify that the OFA synthesized at the FeCl_3_/FA ratio of 9/1 has the maximum thermal stability likely due to its highest aromaticity or C/H ratio. Note that the OFA possesses the highest char yield of up to 77.6 wt% at 1100 °C in argon, which supports the discotic molecular structure of OFA with high thermal stability and high carbonized yield, as opposed to a linear chain structure with relatively low thermal stability and low carbonized yield. Note that the thermal stability of the OFAs synthesized in this study is much higher than that of electropolymerized FA.^10^

**Fig. 9:**
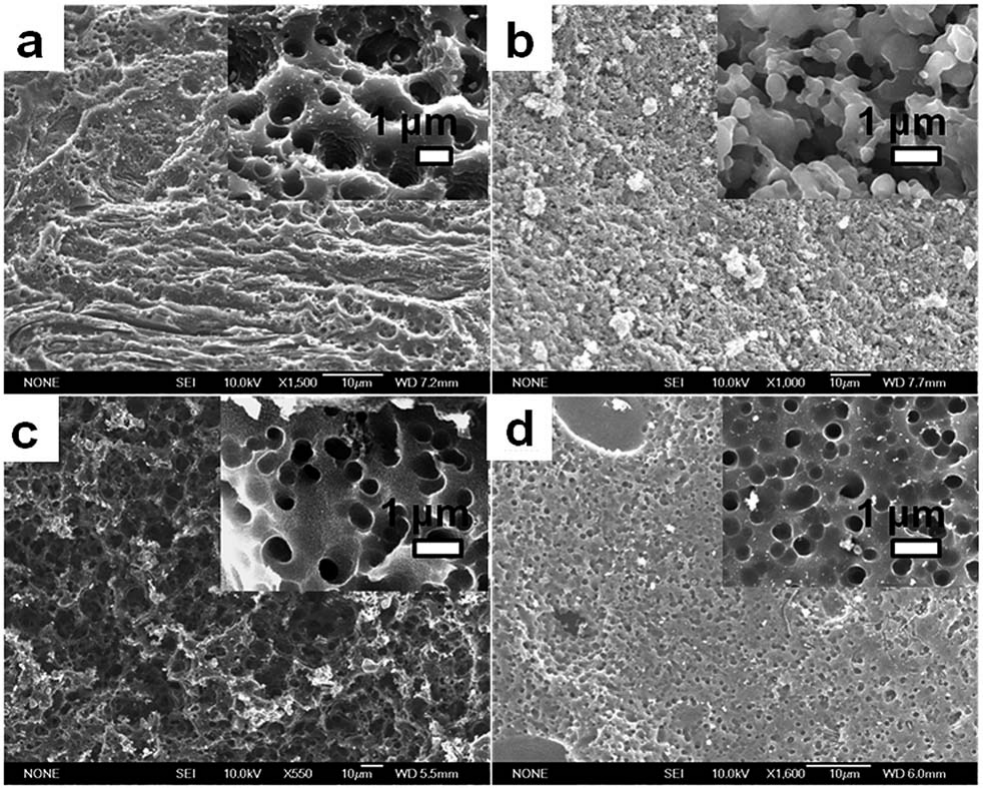
SEM images of porous carbon materials based on the OFAs synthesized with the four FeCl_3_/FA molar ratios: (a) 5/1, (b) 9/1, (c) 12/1, and (d) 15/1.

The high char yield of the OFAs provides a new route to prepare carbon materials at low cost. Fig. 6b shows the XRD curves of four OFAs-based carbon materials made in argon at 1100 °C, demonstrating only one broad diffraction centered at 2*θ* = 23.5° corresponding to the (002) reflection of turbostratic graphite. Meanwhile, a new weak diffraction centered at 2*θ* = 43.5° emerges that can be assigned to the (101) Bragg reflection of graphite.^42^ The carbonized material possesses completely different XRD characteristics from virgin OFA powder when used as a carbon precursor. This implies that the OFAs have been converted into graphite-like carbon materials after thermal pyrolysis in argon at 1100 °C. This result has been confirmed by two other methods: (1) the Raman spectrum of carbonized polyfluoranthene (PFA) at 1100 °C under an inert atmosphere exhibits the two Raman bands expected for carbon: these are the strongest and sharpest bands at 1306 and 1584 cm^–1^ assigned to D- and G-bands, respectively^31^ and (2) much higher electrical conductivity of up to 100 S cm^–1^ of this carbonized OFA when compared to virgin OFA and I_2_-doped OFA powders, as discussed below. SEM images reveal that the obtained carbon materials contain 3D interconnected macropores with diameters ranging from 400 to 1200 nm (Fig. 9).

The OFA possesses a special molecular structure consisting of one naphthalene and benzene bridged by a penta-carbocyclic ring, possibly resulting in some gaps in its supramolecular aggregation, thus leading to porous structures after carbonization. In particular, the macroporous carbon materials have electrical conductivities up to 100 S cm^–1^, which is much higher than that of virgin OFA and I_2_-doped OFA powders. Such a large enhancement of the conductivity can also be attributed to the satisfactory conversion of the OFA into graphitic carbon.

### Fluorescence

**Fig. 10:**
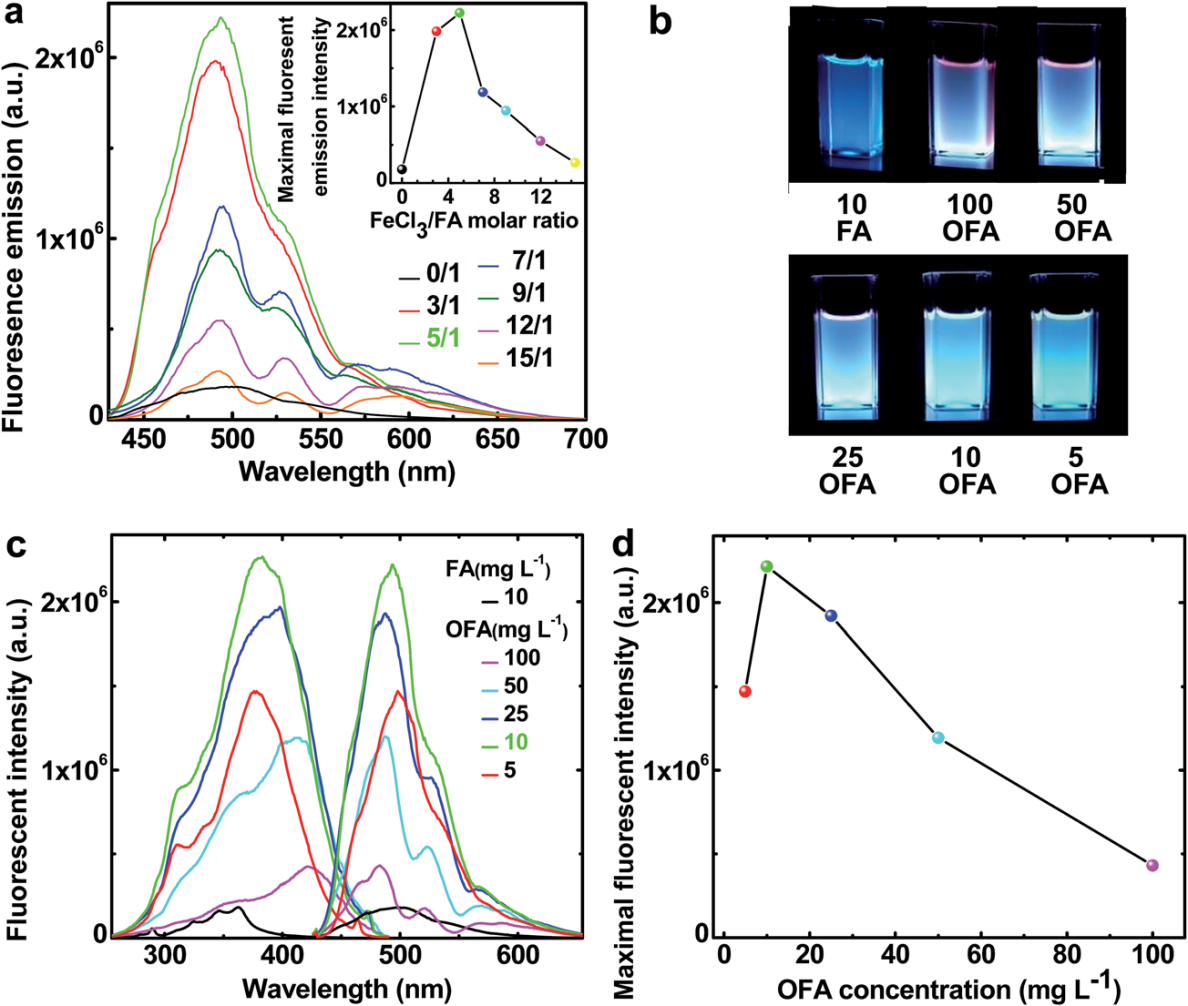
(a) Fluorescence emission (excited @ 395 nm) of FA and the OFAs at the same concentration of 10 mg L^–1^ synthesized with various FeCl_3_/FA molar ratios. Inset: the variation of the maximum fluorescent intensity of FA monomer and OFA solutions in NMP with various FeCl_3_/FA ratios. (b) The images of the fluorescence emission (excited @ 365 nm) of FA and the OFA solutions at different concentrations in NMP. (c) The variation of the excitation (emit @ 460, 483, 490, 490, 490, 490 nm, respectively) and emission fluorescence (excited @ 365, 420, 416, 400, 395, 395 nm, respectively) spectra of FA and OFA solutions in NMP with their concentrations. (d) Maximum fluorescent intensity of OFA solutions at different concentrations in NMP.

The fluorescence emission spectra in NMP of FA and OFAs synthesized with different FeCl_3_/FA ratios are illustrated in Fig. 10a.^8^ By changing the FeCl_3_/FA molar ratio from 0/1 to 15/1, the fluorescence emission first quickly increases and then decreases, as shown in the inset to Fig. 9a, *i.e.*, the fluorescence emission can be adjusted by regulating the FeCl_3_/FA ratio. Most significantly, the OFA synthesized with an FeCl_3_/FA ratio of 5/1 displays the maximum emission fluorescence at 494 nm, possibly due to its symmetric molecular structure as shown in Table 1 and thus the optimal π-conjugation shown in Fig. 1c. The fluorescence excitation/emission intensity can also be tuned by adjusting the OFA concentration. It is found from Fig. 10b–d that 10 mg L^–1^ OFA in NMP demonstrates both the strongest excitation and emission fluorescence at 383 and 494 nm, respectively. Apparently, the OFAs demonstrate different characteristics in their excitation and emission spectra from FA. The bandwidth of the emission spectrum of the OFA is about 70 nm and basically displays a single cyan color at 10 mg L^–1^ as shown in Fig. 10b instead of the blue color of FA. More importantly, the intensity of both the emission and excitation fluorescence is dramatically enhanced by 12.2 times upon oligomerization of FA (Fig. 10c) under exactly the same conditions. Besides the OFA solution, a freestanding robust composite film containing 0.76 wt% OFA in a non-fluorescent polysulfone matrix emits very strong fluorescence when exposed to 365 nm UV light and displays a 4 nm redshift in its emission spectrum similar to the OFA solution.^8^ Strong and controllable fluorescence emission is an important reason why optimized OFA has been shown to be an ultra-sensitive fluorescent emitter for use in amplified and reversible quenching fluorescence chemosensors.^8^,^43^–^50^

## Conclusions

Dispersible OFA nanorods with diameters of 35–40 nm and lengths of >300 nm have been successfully synthesized by a simple template-free oxidative oligomerization of FA in CH_3_NO_2_ using FeCl_3_ as the oxidant. The pentamerization is thought to proceed *via* a cationic oxidative mechanism. The proper oligomerization conditions including oxidant species, oxidant/monomer ratio, polymerization temperature and time have been elucidated for the productive synthesis of OFA with optimal molecular and large π-conjugated structures, morphology, solvatochromism, electrical conductivity, fluorescence, and thermal stability. The OFAs are most likely a five FA-unit-containing cyclic pentameric oligomer that possesses reversible and controllable variation of the electrical conductivity from 10^–11^ S cm^–1^ to 10^–4^ S cm^–1^ by I_2_ doping or dedoping. The OFA nanorods are also excellent precursors to make macroporous carbon materials with pore diameters of 400–1200 nm, carbon yield of 77.6% at 1100 °C, and conductivities up to 100 S cm^–1^. These features make the OFAs of interest as conductivity controllable materials and as high carbon-yield precursors for carbon nanomaterials. Since the emitting fluorescence of the optimized OFA nanorods is 12.2 times stronger than that of recognized highly fluorescent FA of the low molecular organic fluorescent substances, OFAs may have potential as ultrasensitive fluorescent emitters.

## Supplementary Material

Supplementary information
