# Supplementary material for: Efficient synthesis of oligofluoranthene nanorods with tunable functionalities
Source: Chem Sci. 2015 Sep 17;6(12):7190–200. doi: 10.1039/c5sc03041b (PMC5951197; doi:10.1039/c5sc03041b)
Supplement: Supplementary file 1 [file SC-006-C5SC03041B-s001.pdf]

Electronic Supplementary Material (ESI) for Chemical Science.
This journal is © The Royal Society of Chemistry 2015

Chemical Science

2015, 6, DOI: 10.1039/c5sc03041b

Electronic supplementary information

Efficient synthesis of oligofluoranthene nanorods with tunable functionalities

Xin-Gui Li,[‡*ab] Yaozu Liao,[‡abc] Mei-Rong Huang[*ab] and Richard B. Kaner[*bd]

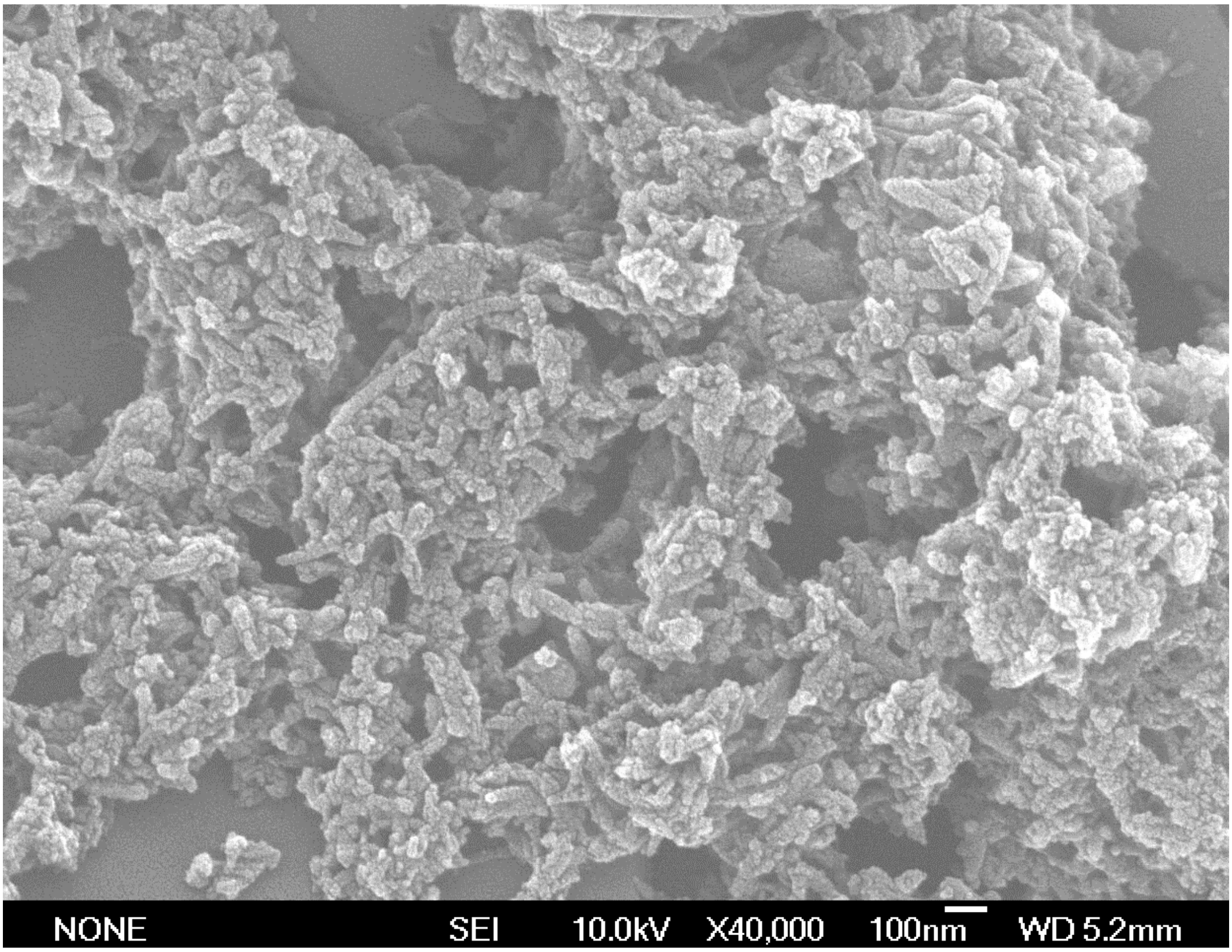

(a) $FeCl_3$/FA molar ratio: 5/1

----

[a]*State Key Laboratory of Pollution Control and Resource Reuse, College of Environmental Science and Engineering, Tongji University, Shanghai 200092, China. E-mail: adamxgli@yahoo.com; huangmeirong@tongji.edu.cn.*

[b]*Department of Chemistry & Biochemistry, California NanoSystems Institute, University of California, Los Angeles, Los Angeles, California 90095, USA. E-mail: kaner@chem.ucla.edu; Fax: +1 310 206 4038; Tel: +1 310 825 5346.*

[c]*State Key Laboratory for Modifi cation of Chemical Fibers and Polymer Materials, College of Materials Science and Engineering, Donghua University, Shanghai 201620, P. R. China*

[d]*Department of Materials Science & Engineering, University of California, Los Angeles, Los Angeles, California 90095, USA*

‡ *X.-G. Li and Y.Z. Liao contributed equally to this work.*

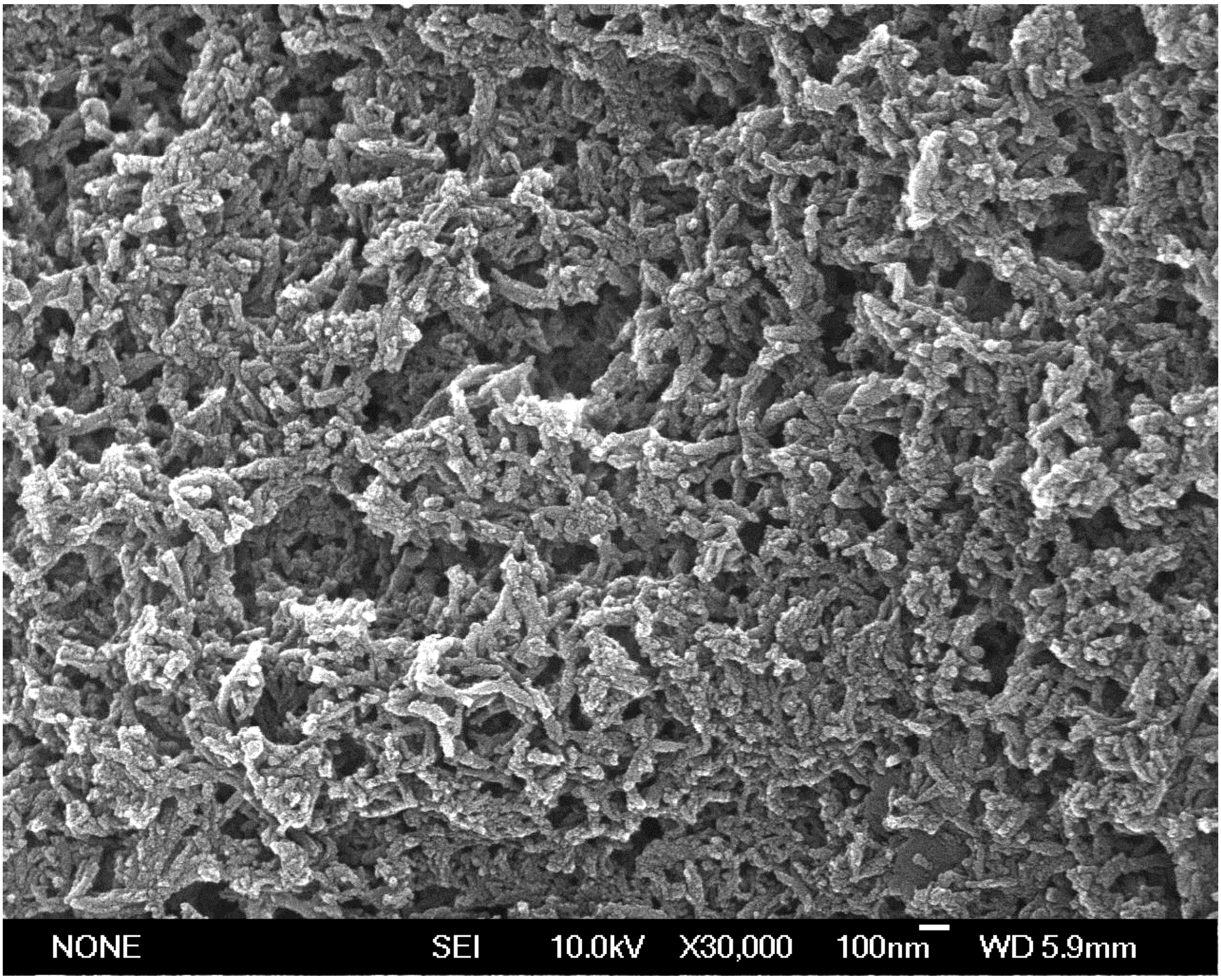

(b) $FeCl_3$/FA molar ratio: 9/1

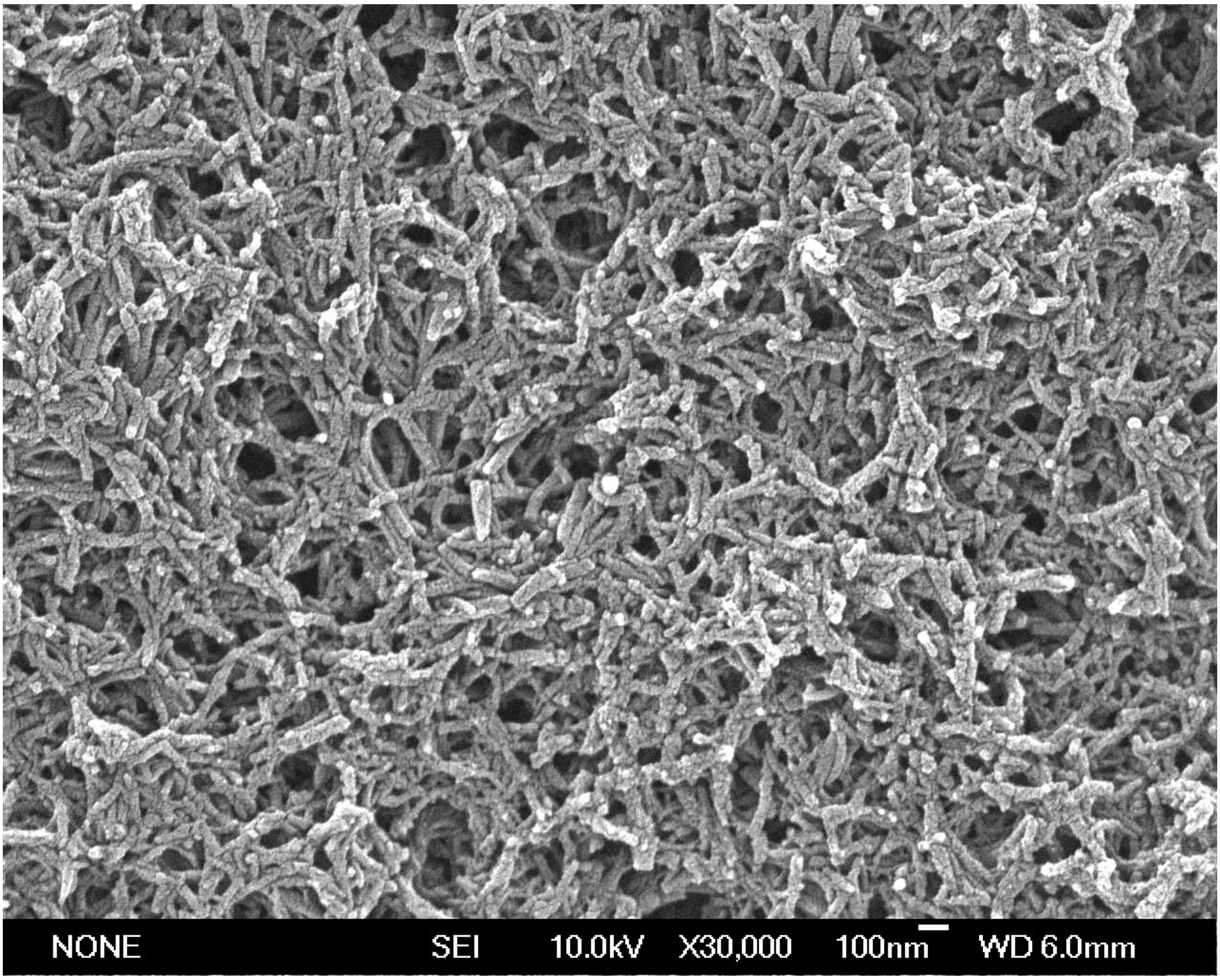

(c) $FeCl_3$/FA molar ratio: 12/1

Figure S1. SEM images of OFA nanorods synthesized with $FeCl_3$/FA molar ratios: (**a**) 5/1, (**b**) 9/1, and (**c**) 12/1.
